# Long-term dietary intervention with low Phe and/or a specific nutrient combination improve certain aspects of brain functioning in phenylketonuria (PKU)

**DOI:** 10.1371/journal.pone.0213391

**Published:** 2019-03-15

**Authors:** Vibeke M. Bruinenberg, Danique van Vliet, Els van der Goot, Danielle S. Counotte, Mirjam Kuhn, Francjan J. van Spronsen, Eddy A. van der Zee

**Affiliations:** 1 Molecular Neurobiology, GELIFES, University of Groningen, Groningen, The Netherlands; 2 Division of Metabolic Diseases, Beatrix Children’s Hospital, University Medical Center of Groningen, University of Groningen, Groningen, The Netherlands; 3 Nutricia Research, Utrecht, The Netherlands; University of Florida, UNITED STATES

## Abstract

**Introduction:**

In phenylketonuria (PKU), a gene mutation in the phenylalanine metabolic pathway causes accumulation of phenylalanine (Phe) in blood and brain. Although early introduction of a Phe-restricted diet can prevent severe symptoms from developing, patients who are diagnosed and treated early still experience deficits in cognitive functioning indicating shortcomings of current treatment. In the search for new and/or additional treatment strategies, a specific nutrient combination (SNC) was postulated to improve brain function in PKU. In this study, a long-term dietary intervention with a low-Phe diet, a specific combination of nutrients designed to improve brain function, or both concepts together was investigated in male and female BTBR PKU and WT mice.

**Material & methods:**

48 homozygous wild-types (WT, +/+) and 96 PKU BTBR^Pah2^ (-/-) male and female mice received dietary interventions from postnatal day 31 till 10 months of age and were distributed in the following six groups: high Phe diet (WT C-HP, PKU C-HP), high Phe plus specific nutrient combination (WT SNC-HP, PKU SNC-HP), PKU low-Phe diet (PKU C-LP), and PKU low-Phe diet plus specific nutrient combination (PKU SNC- LP). Memory and motor function were tested at time points 3, 6, and 9 months after treatment initiation in the open field (OF), novel object recognition test (NOR), spatial object recognition test (SOR), and the balance beam (BB). At the end of the experiments, brain neurotransmitter concentrations were determined.

**Results:**

In the NOR, we found that PKU mice, despite being subjected to high Phe conditions, could master the task on all three time points when supplemented with SNC. Under low Phe conditions, PKU mice on control diet could master the NOR at all three time points, while PKU mice on the SNC supplemented diet could master the task at time points 6 and 9 months. SNC supplementation did not consistently influence the performance in the OF, SOR or BB in PKU mice. The low Phe diet was able to normalize concentrations of norepinephrine and serotonin; however, these neurotransmitters were not influenced by SNC supplementation.

**Conclusion:**

This study demonstrates that both a long-lasting low Phe diet, the diet enriched with SNC, as well as the combined diet was able to ameliorate some, but not all of these PKU-induced abnormalities. Specifically, this study is the first long-term intervention study in BTBR PKU mice that shows that SNC supplementation can specifically improve novel object recognition.

## Introduction

The detrimental effects of increased phenylalanine (Phe) concentrations on the brain are clearly visible in the metabolic disorder Phenylketonuria (PKU, OMIM 261600). In this disorder, a mutation in the gene encoding the hepatic enzyme phenylalanine hydroxylase causes a disruption in the conversion of Phe to tyrosine. Consequently, when no restrictions are made in natural protein intake, Phe accumulates in blood and brain and leads to severe cognitive disabilities and epilepsy[1]. Neonatal screening facilitates early introduction of treatment preventing the development of these symptoms. Nonetheless, even patients, who are diagnosed early and treated continuously, experience deficits in cognitive functioning, for instance in processing speed, attention, working memory and social-cognitive functioning [2–5]. This suggests that current treatment strategies still do not address all patient needs.

In the search for new and/or additional treatment strategies, a combination of specific nutrients (SNC) was postulated to ameliorate the functional and neurobiological effects of increased Phe [6]. These specific nutrients are precursors and cofactors for the synthesis of phospholipids through the Kennedy pathway [7]. Using this same SNC, several experiments have been conducted where an increase in brain phospholipid levels was shown, for example in animal models of Alzheimer’s disease [8], Traumatic Brain Injury [9], and in aged rodents [10]. Since the Kennedy pathway enzymes are not fully saturated under normal conditions, providing more of the precursors will lead to enhanced phospholipid synthesis [11]. The neuronal membrane is rich in phospholipids and we postulate that enhancing phospholipid synthesis could improve some of the neurobiological alterations in PKU, such as synaptic changes and neurotransmitter abnormalities. Indeed, we previously showed that SNC can normalize PSD-95 immunostaining intensity in the PKU mouse brain [6]

It is unclear in which period SNC supplementation could have a beneficial effect for PKU patients using life-long treatment with a Phe restricted diet. In addition, at present little is known about what happens to the brain of early treated PKU patients with aging, as the treatment was implemented just 50–60 years ago. To obtain more insight into the aging PKU brain on a low Phe diet, combined with the beneficial effects of SNC supplementation over time on cognitive and motor behavior, the BTBR PKU mouse model was chosen, as these mice show clear differences in motor function and cognition compared to their wild-type controls [12].

The aim of this study in PKU mice treated from one to ten months of age was thus twofold: 1) to investigate the consequences of a long-term Phe-restricted dietary treatment, and 2) to examine the effect of SNC on the behavioral performance and neurotransmitter concentrations of PKU mice under high Phe and low Phe conditions.

## Methods

### Animals

A breeding colony of heterozygous (+/-) mating pairs generated 48 wild-types (WT, +/+) and 96 PKU BTBR^Pah2^ (-/-) male and female mice. Original breeding pairs were kindly provided by prof. Puglisi-Allegra from the Sapienza, University of Roma, Rome, Italy. Breeding pairs consisted of one male and one female housed together for 14 days. After these 14 days the male was removed from the cage. On postnatal day (PND) 28 the animals were weaned, and the genetic status of the animals was established via quantitative PCR analysis on DNA extracted from ear tissue [12]. After weaning, all littermates were kept in the initial cage (26x42x15, plexiglass) without the mother until PND 31. On PND 31, the animals were group housed in sex-matched pairs of two in cages of 26x42x15 (plexiglass) with sawdust bedding and cage enrichment in the form of nesting material, paper rolls, and a small wooden stick made of Aspen (Abedd). To reduce the chance of fighting among the males, a red transparent house-shaped shelter was added in their cages in the adult stage. The climate condition of the housing facility was kept constant at a temperature of 21±1°C, 51% ±5 humidity, and a 12/12 light/dark cycle. The animals received fresh food every day (between Zeitgeber Time (ZT) 8 and ZT10) and had *ad libitum* access to water. Leftover food was collected every day before fresh food was provided. Together with a thorough search of bedding after cage cleaning, the difference with offered food gave an estimate of weekly food intake per pair. Furthermore, body weight of the mice was measured during this weekly cage cleaning (ZT 8–10). The dietary intervention started at PND 31 until 10 months of age. The length of the experiment required clear humane endpoints. These were set as a decrease in body weight of 15% together with other signs of sickness behavior (e.g. inactive behavior, or displaying an arched back). If one of the pair of mice was excluded from the experiment, females were placed in pairs of three. Males were kept solitary. All experimental procedures were approved by an independent ethics committee for animal experimentation (Animal Ethics Committee of the University of Groningen, permit 6504E, Groningen, the Netherlands) and complied with the principles of good laboratory animal care following the European Directive for the protection of animals used for scientific purposes.

### Dietary intervention

The dietary intervention started on PND 31. At this time point, pairs of mice were assigned to one of the following six groups: WT control high Phe diet (WT C-HP), WT high Phe diet plus SNC (WT SNC-HP), PKU high Phe diet (PKU C-HP), PKU high Phe diet plus SNC (PKU SNC-HP), PKU low-Phe diet (PKU C-LP), and PKU low-Phe diet plus SNC (PKU SNC-LP). The specifics of the diet containing the nutrient combination SNC are depicted in Table 1. Phospholipids in this diet are derived from soy lecithin, namely Emulpur (Cargill Texturizing Solutions, The Netherlands). Emulpur is de-oiled soy crude lecithin and mainly contains phospholipids (77 g phospholipids/100 g lecithin) of which mainly PtdCho (20 g/100 g lecithin), PtdIns (14 g/100 g lecithin), and PtdEtn (13 g/100 g lecithin, as provided by the supplier), with mainly linoleic acid (18:2n-6), palmitic acid (16:0), and oleic acid (18:1n-9).

**Table 1:** Nutritional content of experimental diets.

	C-HP		SNC-HP		C-LP		SNC-LP	
g/100g diet	G	M	G	M	G	M	G	M
Corn starch	33,45	41,34	30,66	38,55	33,87	41,76	31,08	38,97
Mais dextrine	13,20	15,50	13,20	15,50	13,20	15,50	13,20	15,50
Sucrose	10,00	10,00	10,00	10,00	10,00	10,00	10,00	10,00
Dextrose	10,00	5,00	10,00	5,00	10,00	5,00	10,00	5,00
Fiber	5,00	5,00	5,00	5,00	5,00	5,00	5,00	5,00
Alanine	0,46	0,33	0,46	0,33	0,46	0,33	0,46	0,33
Arginine	0,64	0,45	0,64	0,45	0,64	0,45	0,64	0,45
Aspartic acid	1,22	0,80	1,22	0,80	1,22	0,80	1,22	0,80
Cystine	0,37	0,24	0,37	0,24	0,37	0,24	0,37	0,24
Glutamic acid	3,63	2,55	3,63	2,55	3,63	2,55	3,63	2,55
Glycine	0,32	0,23	0,32	0,23	0,32	0,23	0,32	0,23
Histidine	0,46	0,33	0,46	0,33	0,46	0,33	0,46	0,33
Isoleucine	0,82	0,59	0,82	0,59	0,82	0,59	0,82	0,59
Leucine	1,57	1,09	1,57	1,09	1,57	1,09	1,57	1,09
Lysine	1,63	0,92	1,63	0,92	1,63	0,92	1,63	0,92
Methionine	0,46	0,33	0,46	0,33	0,46	0,33	0,46	0,33
**Phenylalanine**	0,62	0,62	0,62	0,62	0,20	0,20	0,20	0,20
Proline	2,05	1,43	2,05	1,43	2,05	1,43	2,05	1,43
Serine	0,97	0,67	0,97	0,67	0,97	0,67	0,97	0,67
Threonine	0,67	0,47	0,67	0,47	0,67	0,47	0,67	0,47
Tryptophan	0,21	0,16	0,21	0,16	0,21	0,16	0,21	0,16
Tyrosine	1,50	1,50	1,50	1,50	1,50	1,50	1,50	1,50
Valine	1,00	0,70	1,00	0,70	1,00	0,70	1,00	0,70
Mineral premix (AIN-93G-MX)		3,5		3,5		3,5		3,5
Mineral premix (AIN-93M-MX)	3,5		3,5		3,5		3,5	
Vitamin premix (AIN-93-VX)	1	1	1	1	1	1	1	1
Soy oil	1,900	1,900			1,900	1,900		
Coconot oil	0,900	0,900	0,100	0,100	0,900	0,900	0,100	0,100
Corn oil	2,200	2,200	1,700	1,700	2,200	2,200	1,700	1,700
DHA25 oil			3,000	3,000			3,000	3,000
EPA28/12 oil			0,200	0,200			0,200	0,200
Choline bitartrate (41,1% choline)	0,250	0,250	0,250	0,250	0,250	0,250	0,250	0,250
Tert-butylhydroquinone	0,0014	0,0014	0,0014	0,0014	0,0014	0,0014	0,0014	0,0014
Pyridoxine-HCL			0,00328	0,00328			0,00328	0,00328
Folic acid (90%)			0,00067	0,00067			0,00067	0,00067
Cyanocobalamin (0,1% in mannitol)			0,00350	0,00350			0,00350	0,00350
Ascorbic acid (100% zuiver)			0,160	0,160			0,160	0,160
dl-α-tocopheryl acetate (500 IU/g)			0,4650	0,4650			0,4650	0,4650
UMP disodium (24%H2O)			1,0	1,0			1,0	1,0
Choline chloride (74,576%)			0,402	0,402			0,402	0,402
Soja lecithine (Emulpur)			0,755	0,755			0,755	0,755
Sodium selenite (46% min)			0,00023	0,00023			0,00023	0,00023
**Energy (kcal/100 g diet)**	**385,99**	**385,99**	**374,84**	**374,84**	**385,99**	**385,99**	**374,84**	**374,84**

The high Phe diet is a normal diet for WT animals. Each group consisted of 12 males and 12 females. As the mice were at the pre-adolescence stage at the beginning of the experiments, the diet was based on the growth diet AIN-93G. In the adult stage (13 weeks), the mice were switched to diets based on the maintenance diet AIN-93M manufactured by the same supplier (Research Diet Services BV, Wijk bij Duurstede, The Netherlands). The key characteristics of the diets were kept the same; normal diet with or without SNC had Phe content of 6.2 g/kg and tyrosine content of 15 g/kg and low-Phe diet (based on previous literature [13]) had Phe content of 2.0 g/kg and tyrosine content of 15 g/kg. During the course of the experiments some animals died, often for unclear reasons. See S1 Table for an overview.

### Behavioral paradigms

**Fig 1:**
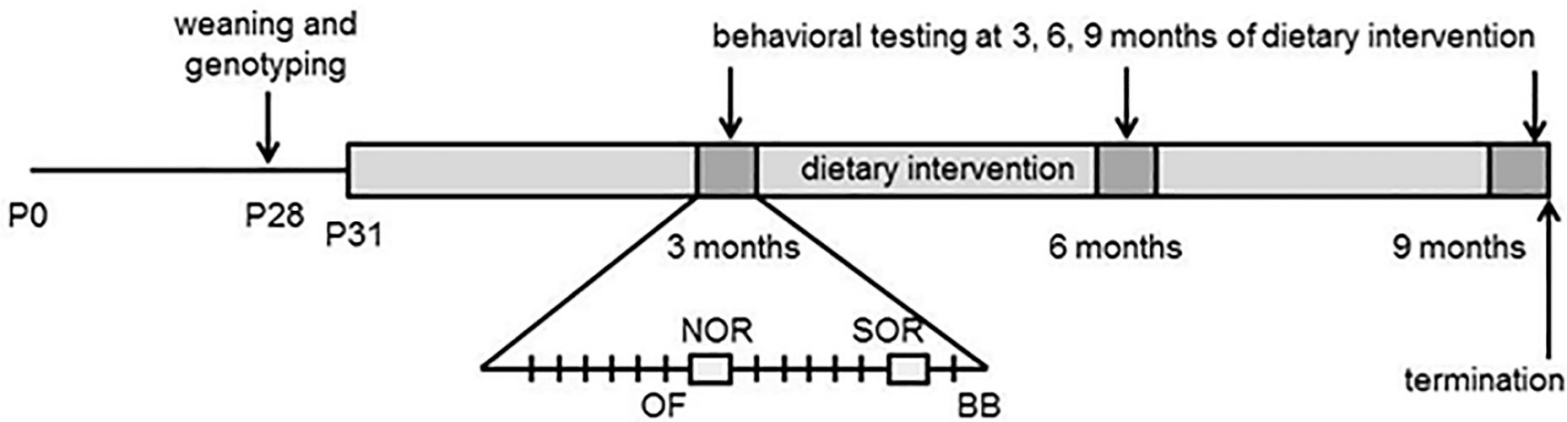
Schematic representation of the experiment.

During the dietary intervention, the animals were behaviorally assessed every 12 weeks starting at four months of age. Each test session consisted of an open field test (OF), novel object recognition (NOR), spatial object recognition (SOR), and a balance beam (BB). These sessions covered a period of 17 days during which only 1 test was conducted on a specific day (see Fig 1 for the testing-scheme). The animals were tested between ZT1and ZT6. All procedures and experimental setups were described in our previous study [12]. In short, the habituation phase of the NOR was used as OF. On day 1 of the testing session, the animals were placed in the middle of a square arena (50x50x35 cm) to explore the arena freely for ten minutes. The subsequent day the animals could explore two identical objects for ten minutes in the familiarization phase of the NOR. Again 24 hours later, one of the objects was replaced with a novel object and the mice could explore this new setting for 10 minutes. After a period of five days without behavioral testing, the animals were tested in the SOR. The first day the animals were exposed to four sessions of 6 minutes. The first session was similar to the habituation phase of the NOR. In the second to the fourth session, the animals could freely explore three different objects (in shape, color, and texture) in a specific configuration. Between sessions, the animals were placed back in the home cage for 2 minutes. The second day, one of the objects was moved to a different location. The objects, the starting condition, and the displaced object were randomized over trials. Both the NOR and SOR were performed in a separate room recorded with a camera (Panasonic WVCP500) connected to a computer outside the room with Media recorder (Noldus, The Netherlands). The balance beam was performed in the housing facility 24 hours later. During this task, the animals had to cross a square wooden beam ((length 1 m, width 5 mm, height 10 mm, horizontally positioned 50 cm above the underlying surface) over four distances (10, 40, 75, and 100 cm). The final distance was used as read-out trial. In this trial, the number of correct steps and total steps necessary to cross the beam were manually scored and calculated to a percentage. A step was considered correct if the hind paw had a full placement on the beam at the initiation and end of the forward movement.

The open field was analyzed with Ethovision v.11 (Noldus, The Netherlands). In this analysis, the arena was divided into a center zone, four border zones, and four corner zones [14]. Activity was quantified by the distance moved, and anxiety-like behavior was examined by the preference of the animal to visit, or stay in, the more sheltered zones, i.e. the corners. In the NOR and SOR, the exploration time of each object was manually scored with the program ELINE (made in house). For the NOR, the discrimination index (DI) was calculated by the time spent exploring the novel object minus the time exploring the same object divided by the total exploration time of both objects [15]. For the SOR, the exploration time of the first three training sessions was compared to the time exploring in the test session. The mice mastered these learning paradigms when they explored either the novel object or the relocated object above chance level.

### Neurotransmitter

At the end of the experiment, all mice were euthanized and tissue was collected (procedures were performed between ZT5 and ZT7). After deeply anesthetizing the animals with isoflurane, the mice were briefly perfused with 0,9% NaCl, 0.1% EDTA solution. The brain, without cerebellum and brainstem, was flash frozen in liquid nitrogen and stored at the -80°C. Whole brains of twelve animals (6 males/ 6 females) of each group (pairs were randomly chosen) were used to determine neurotransmitter concentrations using liquid chromatography in combination with isotope dilution mass spectrometry, as previously described [16]. We used whole brain homogenates as we did not expect to find significant regional effects of SNC in certain parts of the brain for neurotransmitter levels. SNC will affect all brain areas containing neurotransmitter producing neurons (e.g. serotonin and norepinephrine in this study) and in our PKU mice whole brain homogenates showed strong reductions in neurotransmitter levels indicating that is a sensitive approach to determine changes in neurotransmitter level [17].

### Statistics

All statistical analyses were performed with SPSS 22.0 (SPSS Inc. SPSS for Windows, Version 22.0. Chicago, SPSS Inc.). Food intake was tested non-parametrically with a Kruskal-Wallis and *post hoc* analysis was done with a Mann-Whitney U test. Body weight and behavioral paradigms have been analyzed using two mixed-effect model repeated measure (MMRM) models. As the groups were not fully balance between the genotypes (WT mice are not able to receive low Phe diet, as reduced intake of an essential amino acid is harmful for the animals), two models were tested: 1) Differences between WT and PKU mice on high Phe diet and the influence of SNC supplementation within these groups were tested using an MMRM model with time, genotype and the interaction term as factors, 2) Differences between the four different diets in PKU mice (C-HP, SNC-HP, C-LP, and SNC-LP) were tested using an MMRM model with time, specific nutrient combination and Phe condition. For body weight, we assume that the body weight measurements taken close to short intervals are more closely related to each other than the measurements taken with a larger time interval (for example we expect that the body weight measurement of week 13 is more similar to week 14 than week to 41). Therefore, the repeated covariance type was set to first order autoregressive. For the behavioral data this assumption was not made, therefore a diagonal covariance type was selected. Checks for normality (Shapiro-Wilk test) and homogeneity of variance (Levene’s test) have been performed.

Furthermore, the ability to master the task was investigated by comparing the DI to chance level (0) with a t-test. No corrections were made for multiplicity. Finally, an ANOVA with factors for sex and group with a Bonferroni correction was used to examine differences between groups in neurotransmitter concentrations. A two-sided p-value equal to or less than 0.05 was considered significant. If not specified, data are expressed as mean ± standard error of the mean (SEM).

## Results

### General health, body weight, and food intake

In the course of the experiment, 21 of the in total 144 animals were excluded from the experiment, because they reached a humane endpoint or died before the end of the study. The excluded animals (see S1 Table) were not in a specific treatment group (Kruskal-Wallis test, groups p = 0.081) and dropout was not skewed by genotype or sex (Kruskal-Wallis test, genotype p = 0.134, sex p = 0.480).

**Fig 2:**
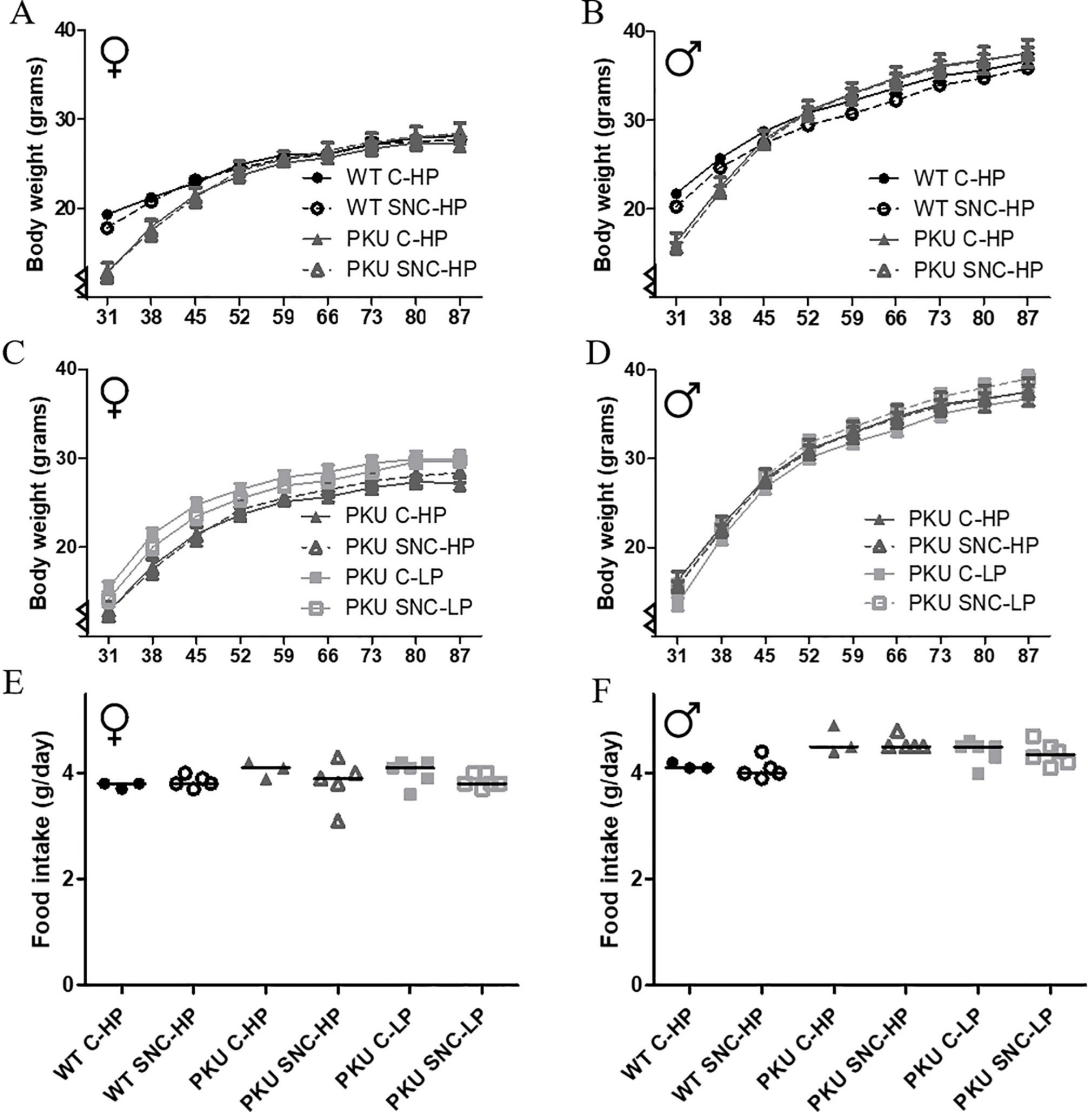
Growth diet. Results are separated for females and male (graphs A,C,E and graphs B,D,F, respectively). In figure A and B, the bodyweight curves of the first eight weeks of treatment, starting on postnatal day 31, are depicted for all groups on high Phe diet (WT C-HP, WT SNC-HP, PKU C-HP, PKU SNC-HP). In figure C and D, the body weight curves for all PKU mice groups are depicted. Mean daily food intake is depicted in graph E and F (median depicted). Graphs A-D: mean ± SEM, x-axis depict days. Graphs E-F: median.

**Fig 3:**
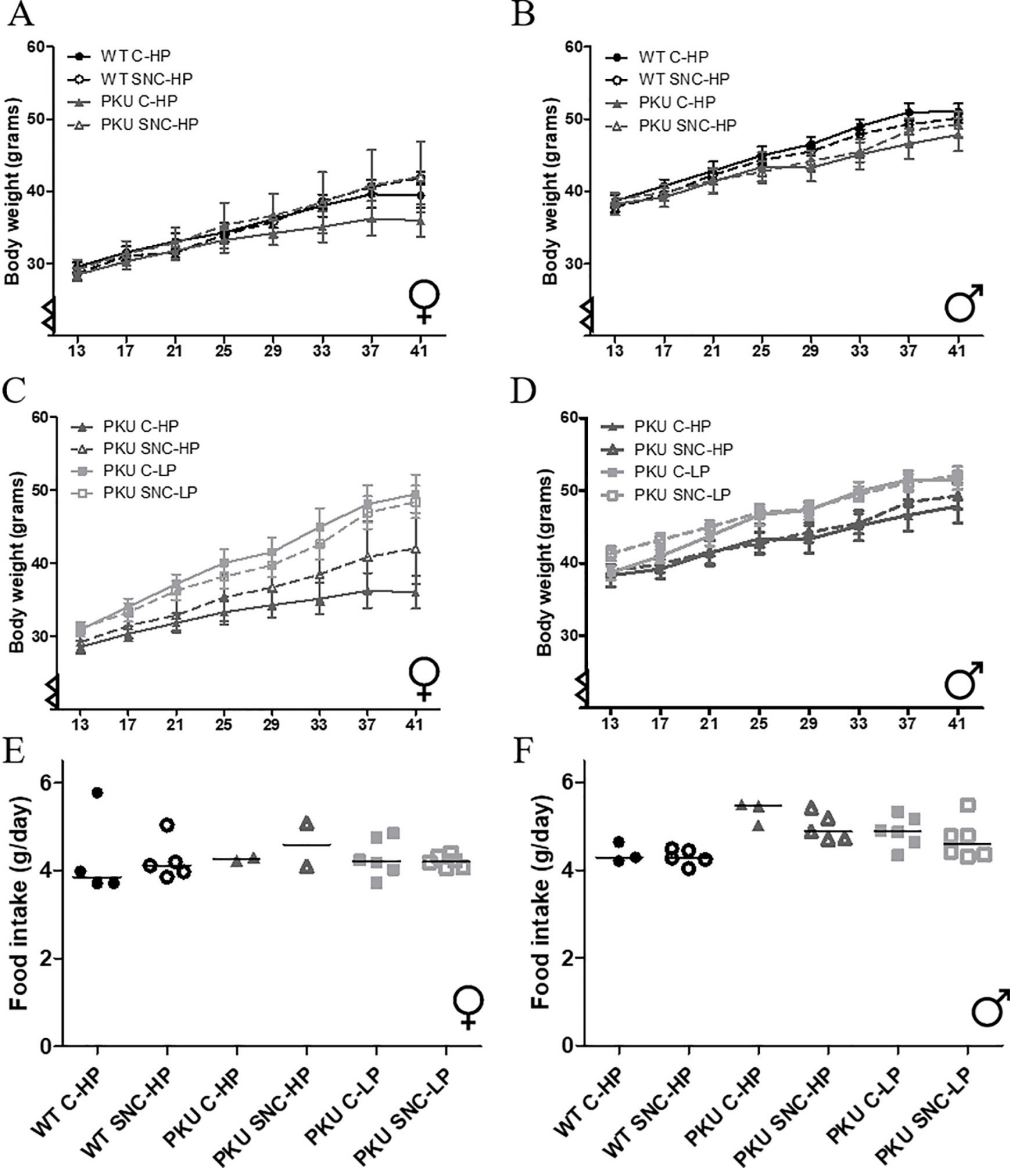
Maintenance diet. Graphs are identically organized as Fig 2. In graphs A-D, the bodyweight curves of the last 28 weeks of dietary treatment are depicted, starting at week 13. In graphs E-F, mean daily food intake is depicted. Graphs A-D: mean ± SEM, x-axis depict weeks. Graphs E-F: median.

The general health of the animals was, among others, monitored by body weight and food intake. Both parameters were split for growth diet and maintenance diet: the first 9 weeks (Fig 2) and starting from 3 months (Fig 3) respectively. Furthermore, males and females were analyzed separately as food intake and body weight was different between the sexes. In addition, graphs and analyses were split for all groups on a high Phe diet (model 1) and all PKU groups (model 2). Overall, PKU mice have a lower body weight immediately after weaning, but catch up in weight with their wild-type controls over time (Fig 2A–2D). Interestingly, SNC has a different effect on body weight increase in females than males when comparing PKU and WT mice on a high Phe diet (Fig 2A and 2B). Specifically, in females, the increase in body weight differed between WT and PKU mice (genotype x time p<0.001), and an interaction was found between genotype and SNC supplementation (genotype x time x SNC p = 0.005), which was not present in males, (time p<0.001, genotype x time p<0.001, genotype x time x SNC p = 0.871). When transferred to maintenance diet at 3 months of age, in male mice the differences in the increase of body weight over time between WT and PKU on high Phe diet were still present (genotype x time p = 0.002), but in females this interaction was no longer significant (genotype x time p = 0.754). When comparing PKU mice on high and low Phe diet, the increase in body weight over time was different between high Phe and low Phe conditions (Female: Phe condition x time p = 0.002, Male Phe condition x time p = 0.009), but SNC supplementation did not significantly change this.

Despite the difference in body weight gain between the groups, no differences were found in food intake of either the growth or maintenance diet between the groups in female mice (p = 0.173), and in male mice only between WT C-HP and PKU SNC-HP (p = 0.036). However, it is important to note that a mere indication of the differences in food intake can be drawn from these data, as only group housed individuals of the same genotype were included in this analysis.

### Open field

**Fig 4:**
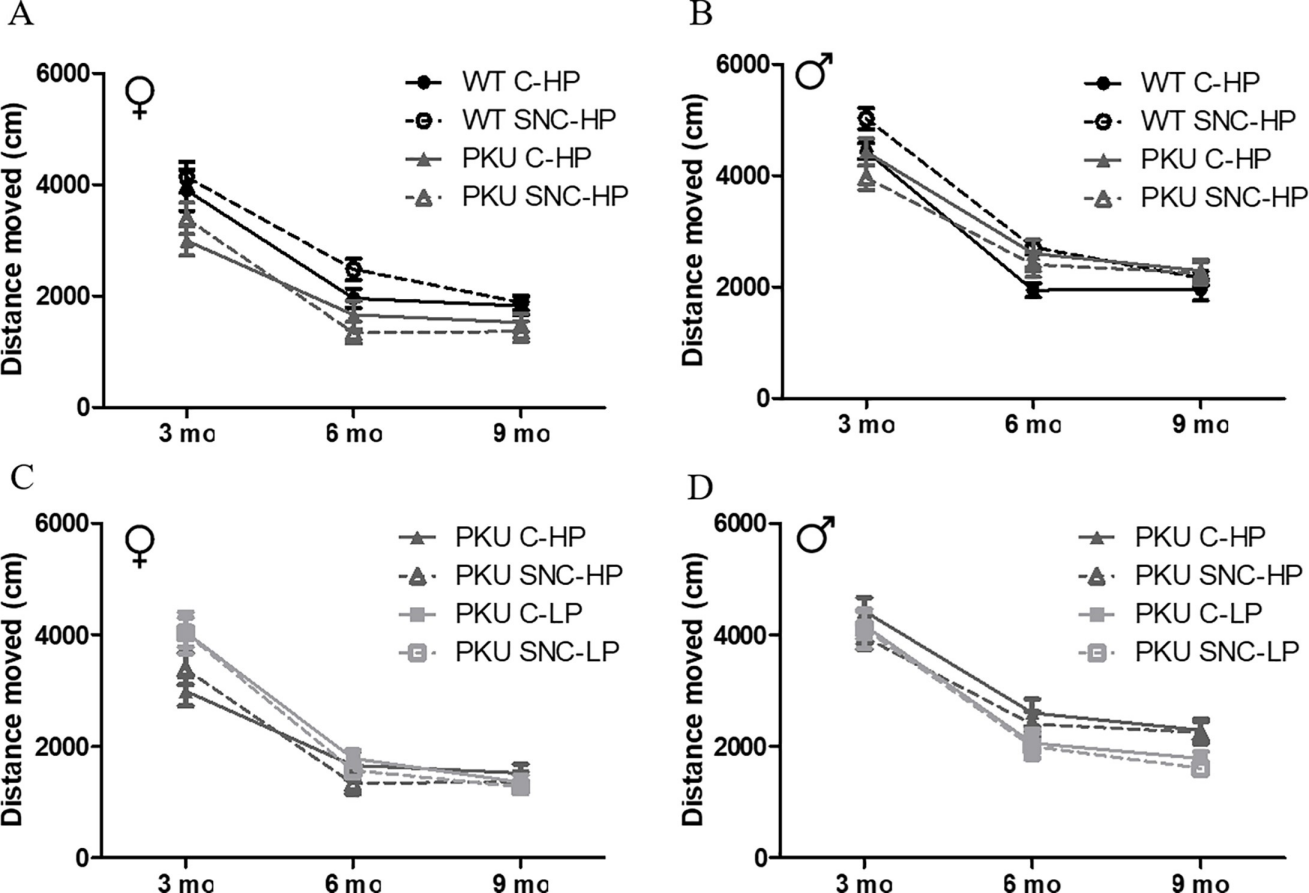
Distance moved in open field. (A) Female mice on high Phe diet WT C-HP (3 mo); n = 11, WT SNC-HP (3mo); n = 12, PKU C-HP (3mo); n = 11, PKU SNC-HP (3mo); n = 9, WT C-HP (6 mo); n = 11, WT SNC-HP (6mo); n = 12, PKU C-HP (6mo); n = 10, PKU SNC-HP (6mo); n = 7, WT C-HP (9 mo); n = 11, WT SNC-HP (9mo); n = 12, PKU C-HP (9mo); n = 10, PKU SNC-HP (9mo); n = 7, (B) male mice on high Phe diet, WT C-HP (3 mo); n = 12, WT SNC-HP (3mo); n = 12, PKU C-HP (3mo); n = 12, PKU SNC-HP (3mo); n = 11, WT C-HP (6 mo); n = 12, WT SNC-HP (6mo); n = 12, PKU C-HP (6mo); n = 12, PKU SNC-HP (6mo); n = 12, WT C-HP (9 mo); n = 12, WT SNC-HP (9mo); n = 9, PKU C-HP (9mo); n = 11, PKU SNC-HP (9mo); n = 9 (C) female PKU mice, PKU C-HP (3mo); n = 11, PKU SNC-HP (3mo); n = 9, PKU C-LP (3mo); n = 12, PKU SNC-LP; n = 12, PKU C-HP (6mo); n = 10, PKU SNC-HP (6mo); n = 7, PKU C-LP (6mo); n = 11, PKU SNC-LP (6mo), n = 11, PKU C-HP (9mo); n = 10, PKU SNC-HP (9mo); n = 7, PKU C-LP (9mo); n = 11, PKU SNC-LP (9mo); n = 11 and (D) male PKU mice, PKU C-HP (3mo); n = 12, PKU SNC-HP (3mo); n = 11, PKU C-LP (3mo); n = 12, PKU SNC-LP (3mo);n = 12, PKU C-HP (6mo); n = 12, PKU SNC-HP (6mo); n = 12, PKU C-LP (6mo); n = 12, PKU SNC-LP (6mo), n = 11, PKU C-HP (9mo); n = 11, PKU SNC-HP (9mo); n = 9, PKU C-LP (9mo); n = 11, PKU SNC-LP (9mo); n = 10. (mean ± SEM).

There were subtle differences in distance moved in the OF between PKU and WT animals. These differences were most pronounced in the female PKU high Phe group compared to their WT counterparts (Fig 4). The distance covered in the open field was examined to explore differences in activity and exploration. Sex differences were observed, and therefore male and female mice were analyzed separately. Clearly, all groups move less through the open field over time because of habituation to the open field. (Fig 4A Female: Time p<0.001; Fig 4B Male: Time p<0.001; Fig 4C Female: Time p<0.001; Fig 4D Male: Time p<0.001). In female mice, the WT mice covered more distance in the maze compared to PKU high Phe mice (p<0.001). In male mice, the progression over time was different between WT and PKU high Phe mice (p = 0.013) but no main effect was found (p = 0.663). The distance moved was differently influenced by SNC supplementation in WT and PKU mice (p = 0.001). In female PKU mice (Fig 3C), PKU mice on low Phe diet covered more distance through the OF compared to PKU mice on high Phe diet (p = 0.022). Furthermore, the progression over time was different between these groups (p = 0.017). In male PKU mice (Fig 4D), the difference between high Phe and low Phe diet was inversed. The mice in the high Phe condition moved more through the open field compared to the low Phe condition (p = 0.006). The progress over the three time points did not differ between the conditions (p = 0.254).

**Fig 5:**
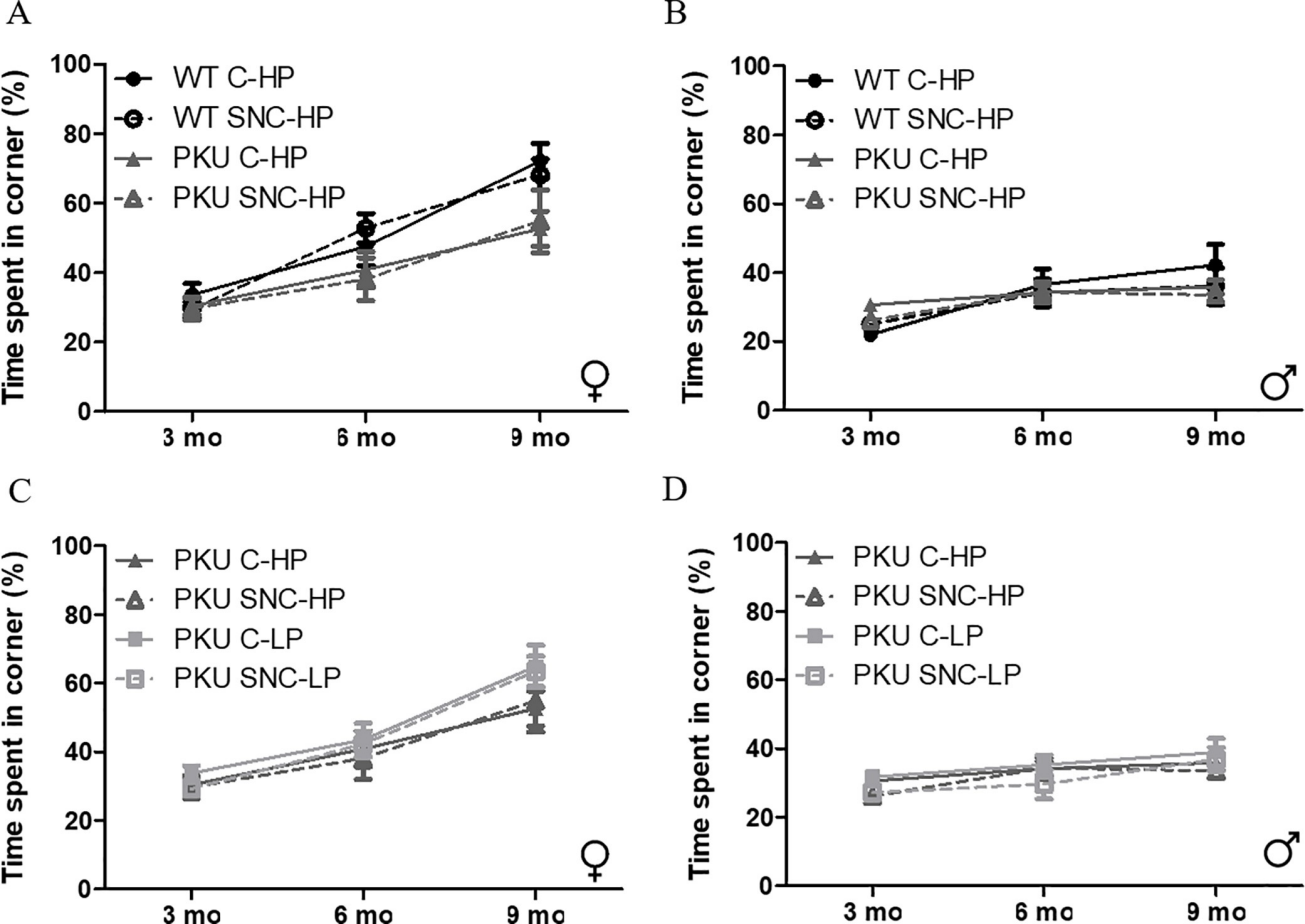
Time spent in corners of the open field. The time spent in the corners of the open field is thought to represent anxiety-like behavior as the mice seek out the more sheltered areas of the arena. (A) Female mice on high Phe diet WT C-HP (3 mo); n = 11, WT SNC-HP (3mo); n = 12, PKU C-HP (3mo); n = 11, PKU SNC-HP (3mo); n = 9, WT C-HP (6 mo); n = 11, WT SNC-HP (6mo); n = 12, PKU C-HP (6mo); n = 10, PKU SNC-HP (6mo); n = 7, WT C-HP (9 mo); n = 11, WT SNC-HP (9mo); n = 12, PKU C-HP (9mo); n = 10, PKU SNC-HP (9mo); n = 7, (B) male mice on high Phe diet, WT C-HP (3 mo); n = 12, WT SNC-HP (3mo); n = 12, PKU C-HP (3mo); n = 12, PKU SNC-HP (3mo); n = 11, WT C-HP (6 mo); n = 12, WT SNC-HP (6mo); n = 12, PKU C-HP (6mo); n = 12, PKU SNC-HP (6mo); n = 12, WT C-HP (9 mo); n = 12, WT SNC-HP (9mo); n = 9, PKU C-HP (9mo); n = 11, PKU SNC-HP (9mo); n = 9 (C) female PKU mice, PKU C-HP (3mo); n = 11, PKU SNC-HP (3mo); n = 9, PKU C-LP (3mo); n = 12, PKU SNC-LP;n = 12, PKU C-HP (6mo); n = 10, PKU SNC-HP (6mo); n = 7, PKU C-LP (6mo); n = 11, PKU SNC-LP (6mo), n = 11, PKU C-HP (9mo); n = 10, PKU SNC-HP (9mo); n = 7, PKU C-LP (9mo); n = 11, PKU SNC-LP (9mo); n = 11 and (D) male PKU mice, PKU C-HP (3mo); n = 12, PKU SNC-HP (3mo); n = 11, PKU C-LP (3mo); n = 12, PKU SNC-LP (3mo);n = 12, PKU C-HP (6mo); n = 12, PKU SNC-HP (6mo); n = 12, PKU C-LP (6mo); n = 12, PKU SNC-LP (6mo), n = 11, PKU C-HP (9mo); n = 11, PKU SNC-HP (9mo); n = 9, PKU C-LP (9mo); n = 11, PKU SNC-LP (9mo); n = 10. (mean ± SEM).

When assessing the innate preference of mice to explore more sheltered areas of the arena, there are clear differences in time spent in the corners between male and female mice, with females increasingly spending more time in the corner over time (Fig 5). Over time, the time spent in the corners was not constant (Fig 5A Female: Time p<0.001; Fig 5B Male; Time p<0.001; Fig 5C Female: Time p<0.001; Fig 5D Male: Time p<0.001). In the females, the PKU high Phe mice spent less time in the corners compared to WT mice (Fig 5A, genotype p<0.001) with less of an increase in time spent in the corner between the three time points (p = 0.040). In male mice, no difference was observed between WT and PKU high Phe mice or the progression over time (genotype = 0.906, time x genotype = 0.055).

### Learning and memory paradigms: NOR and SOR

**Fig 6:**
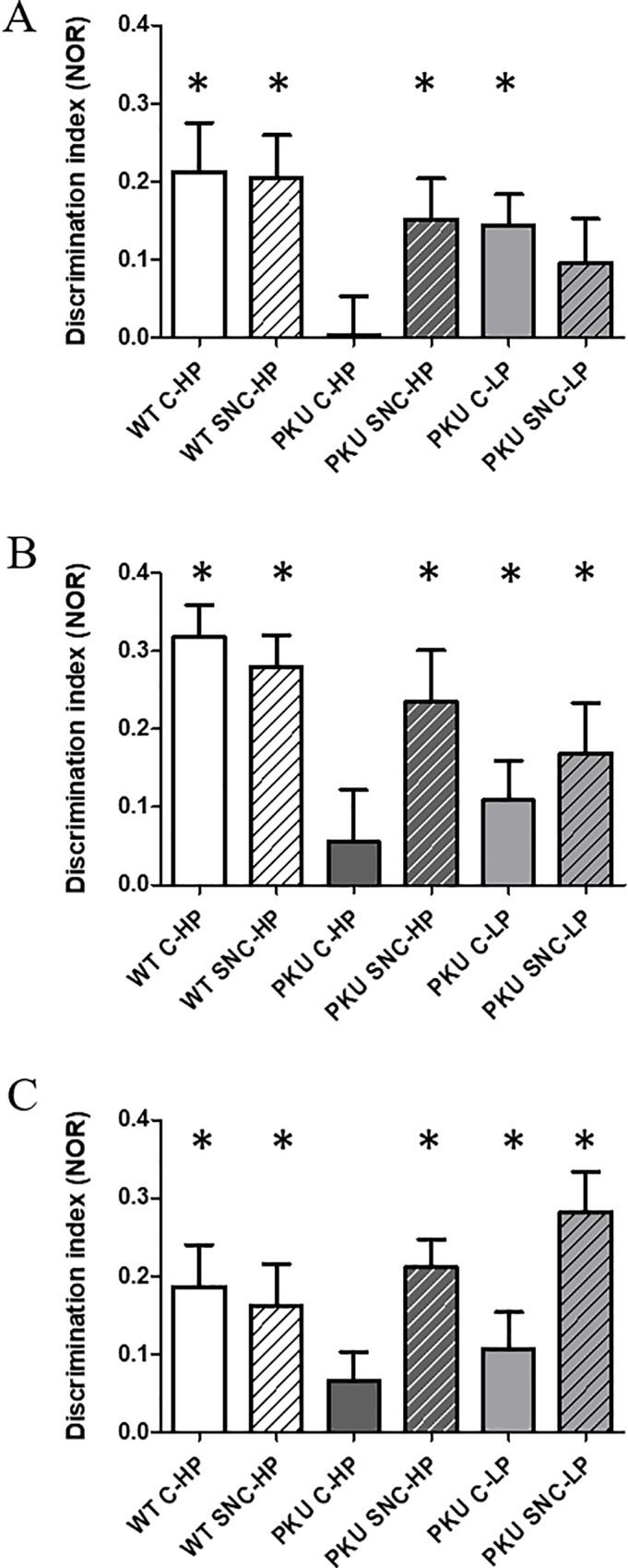
Novel object recognition. Discrimination index ((exploration novel object- exploration same object)/total exploration time) is tested against chance level (0). (A) 3 months, WT C-HP; n = 23, WT SNC-HP; n = 24, PKU C-HP; n = 23, PKU SNC-HP; n = 21,PKU C-LP; n = 24, PKU SNC-HP = 24, (B) 6 months, WT C-HP; n = 23, WT SNC-HP; n = 24, PKU C-HP; n = 22, PKU SNC-HP; n = 19,PKU C-LP; n = 24, PKU SNC-HP = 23 (C) 9 months, WT C-HP; n = 22, WT SNC-HP; n = 21, PKU C-HP; n = 21, PKU SNC-HP; n = 16, PKU C-LP; n = 23, PKU SNC-HP = 21 * represent a significant difference from chance level. (mean ± SEM).

Two separate paradigms were used to assess learning and memory in the PKU and WT mice on different diets (Table 2). Since there were no sex-differences, males and females were analyzed together in these paradigms. Similar to previous findings [12], in the NOR a significant difference was found between WT and PKU high Phe diet in DI p<0.001). SNC supplementation improved the performance of PKU mice (p<0.001) while no overall differences were found for high or low Phe conditions (p = 0.324) nor for the SNC supplementation (p = 0.236). In addition to using this statistical analysis to highlight the differences between the groups, DI was compared to chance level, to assess the ability of the mice to master the learning and memory paradigm. From Fig 6A, it is clear that the mice of the WT-groups (C-HP t(23) = 3.407, p = 0.002, SNC-HP t(23) = 3.715, p = 0.001), PKU SNC-HP (t(20) = 2.915, p = 0.009), and PKU C-LP (t(23) = 3.646 p = 0.001) are able to master the task after three months of treatment. However, the PKU C-HP (t(22) = 0.070 p = 0.944) and PKU SNC-LP (t(23) = 1.707 p = 0.103) did not. After six months of treatment (Fig 6B), all groups except for the PKU C-HP learned the task (WT C-HP t(23) = 7.789 p<0.001, WT SNC-HP t(23) = 6.924 p<0.001, PKU C-HP t(20) = 0.826 p = 0.418, PKU SNC-HP t(18) = 3.573 p = 0.002, PKU C-LP t(23) = 2.137, p = 0.043, PKU SNC-LP t(22) = 2.648 p = 0.015). After the nine months of treatment, for a second time, all groups mastered the NOR task, with the exception of PKU C-HP (WT C-HP t(21) = 3.482 p = 0.002, WT SNC-HP t(20) = 3.081 p = 0.006, PKU C-HP t(21) = 1.729 p = 0.098, PKU SNC-HP t(15) = 6.037 p<0.001, PKU C-LP t(22) = 2.230 p = 0.036, PKU SNC-LP t(20) = 5.461, p<0.001).

**Table 2:** Discrimination index of the SOR. Data are shown as mean ± SEM.

Discrimination index	WT		PKU			
	C-HP	SNC-HP	C-HP	SNC-HP	C-LP	SNC-LP
3 mo–displaced object	6.5 ± 2.7	6.8 ± 2.8	3.4 ± 3.1	5.2 ± 4.4	6.5 ± 3.0	5.0 ± 3.6
3 mo–non displaced object	-3.3 ± 1.3	-2.3 ± 1.4	-0.9 ± 1.6	-1.9 ± 2.4	-3.2 ± 1.5	-1.7 ± 1.9
6 mo–displaced object	7.5 ± 4.0	7.1 ± 4.1	10.6 ± 2.9	4.9 ± 3.0	13.2 ± 3.4	8.4 ± 3.6
6 mo–non displaced object	-3.7 ± 2.0	-3.6 ± 2.1	-5.3 ± 1.4	-2.4 ± 1.5	-6.6 ± 1.7	-4.2 ± 1.8
9 mo–displaced object	3.9 ± 2.4	9.4 ± 2.6	4.3 ± 2.4	10.6 ± 5.8	5.4 ± 4.1	2.2 ± 4.7
9 mo—non displaced object	-2.0 ± 1.2	-4.7 ± 1.3	-2.1 ± 1.2	-5.3 ± 2.9	-2.7 ± 2.0	-1.1 ± 2.4

In PKU mice, SNC supplementation did not affect the time spent on exploring the objects (p = 0.294). PKU mice on high Phe diet did spend more time exploring the objects (p = 0.012). In WT mice, SNC supplementation increased the exploration (p = 0.033).

The analysis of the SOR data did not reveal a PKU phenotype of the PKU control high Phe group compared to the WT group (p = 0.768). However, when comparing the DI to chance level and eliminating outliers (values two standard deviations outside the mean), similar results were found after three months of treatment as described above in the NOR. The mice of the WT-groups (C-HP t(23) = 2.450, p = 0.022, SNC-HP t(22) = 2.123, p = 0.045), PKU SNC-HP (t(19) = 2.228, p = 0.038), and PKU C-LP (t(23) = 2.193 p = 0.037) were able to master the task after three months of treatment, while the PKU C-HP (t(19) = -.069 p = 0.946) and PKU SNC-LP (t(23) = 1.385 p = 0.179) were not. However, the WT mice could no longer master the SOR after six months of treatment (C-HP t(22) = 2.450, p = 0.074, SNC-HP t(23) = 1.719, p = 0.099), leaving no window to observe an improvement with the dietary intervention.

### Balance beam

**Fig 7:**
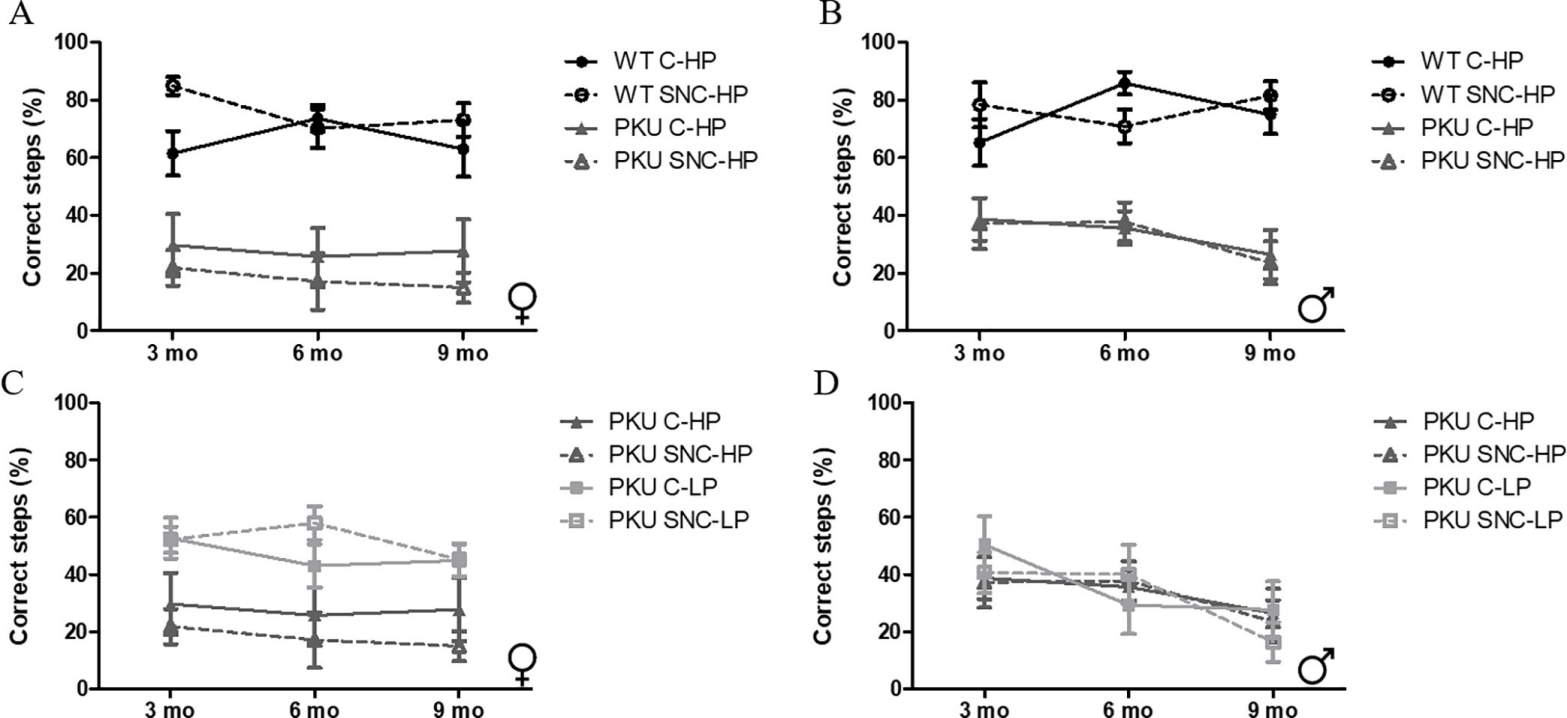
Motor performance. The relative number of correct steps made in the probe trial (100 cm) is depicted. (A) Female mice on high Phe diet, WT C-HP (3 mo); n = 11, WT SNC-HP (3mo); n = 12, PKU C-HP (3mo); n = 11, PKU SNC-HP (3mo); n = 9, WT C-HP (6 mo); n = 11, WT SNC-HP (6mo); n = 12, PKU C-HP (6mo); n = 10, PKU SNC-HP (6mo); n = 7, WT C-HP (9 mo); n = 11, WT SNC-HP (9mo); n = 12, PKU C-HP (9mo); n = 10, PKU SNC-HP (9mo); n = 7 (B) male mice on high Phe diet, WT C-HP (3 mo); n = 12, WT SNC-HP (3mo); n = 12, PKU C-HP (3mo); n = 12, PKU SNC-HP (3mo); n = 11, WT C-HP (6 mo); n = 12, WT SNC-HP (6mo); n = 12, PKU C-HP (6mo); n = 12, PKU SNC-HP (6mo); n = 12, WT C-HP (9 mo); n = 12, WT SNC-HP (9mo); n = 9, PKU C-HP (9mo); n = 11, PKU SNC-HP (9mo); n = 9 (C) female PKU mice, PKU C-HP (3mo); n = 11, PKU SNC-HP (3mo); n = 9, PKU C-LP (3mo); n = 12, PKU SNC-LP;n = 12, PKU C-HP (6mo); n = 10, PKU SNC-HP (6mo); n = 7, PKU C-LP (6mo); n = 11, PKU SNC-LP (6mo), n = 11, PKU C-HP (9mo); n = 10, PKU SNC-HP (9mo); n = 7, PKU C-LP (9mo); n = 11, PKU SNC-LP (9mo); n = 11, and (D) male PKU mice, PKU C-HP (3mo); n = 12, PKU SNC-HP (3mo); n = 11, PKU C-LP (3mo); n = 12, PKU SNC-LP (3mo);n = 12, PKU C-HP (6mo); n = 12, PKU SNC-HP (6mo); n = 12, PKU C-LP (6mo); n = 12, PKU SNC-LP (6mo), n = 11, PKU C-HP (9mo); n = 11, PKU SNC-HP (9mo); n = 9, PKU C-LP (9mo); n = 11, PKU SNC-LP (9mo); n = 10. (mean ± SEM).

Motor balance and coordination was assessed using the BB, In both females and males a clear difference was observed in the percentage of correct steps between WT and PKU mice on high Phe diet (Fig 7A Female: p<0.001;Fig 7B Male: p<0.001). In female PKU mice (Fig 7A), SNC supplementation reduced the relative number of correct steps (p = 0.037), while in male PKU mice, this effect was not observed (p = 0.785).

A low Phe diet was able to improve performance on the balance beam in female, but not male, PKU mice (Fig 7C Female; p<0.001, Fig 7D Male; p = 0.863). When analyzing the overall effect of SNC supplementation in both high and low Phe groups, SNC was not able to change the performance of the PKU mouse groups (female: p = 0.587, male: p = 0.671).

### Neurotransmitters in brain

**Fig 8:**
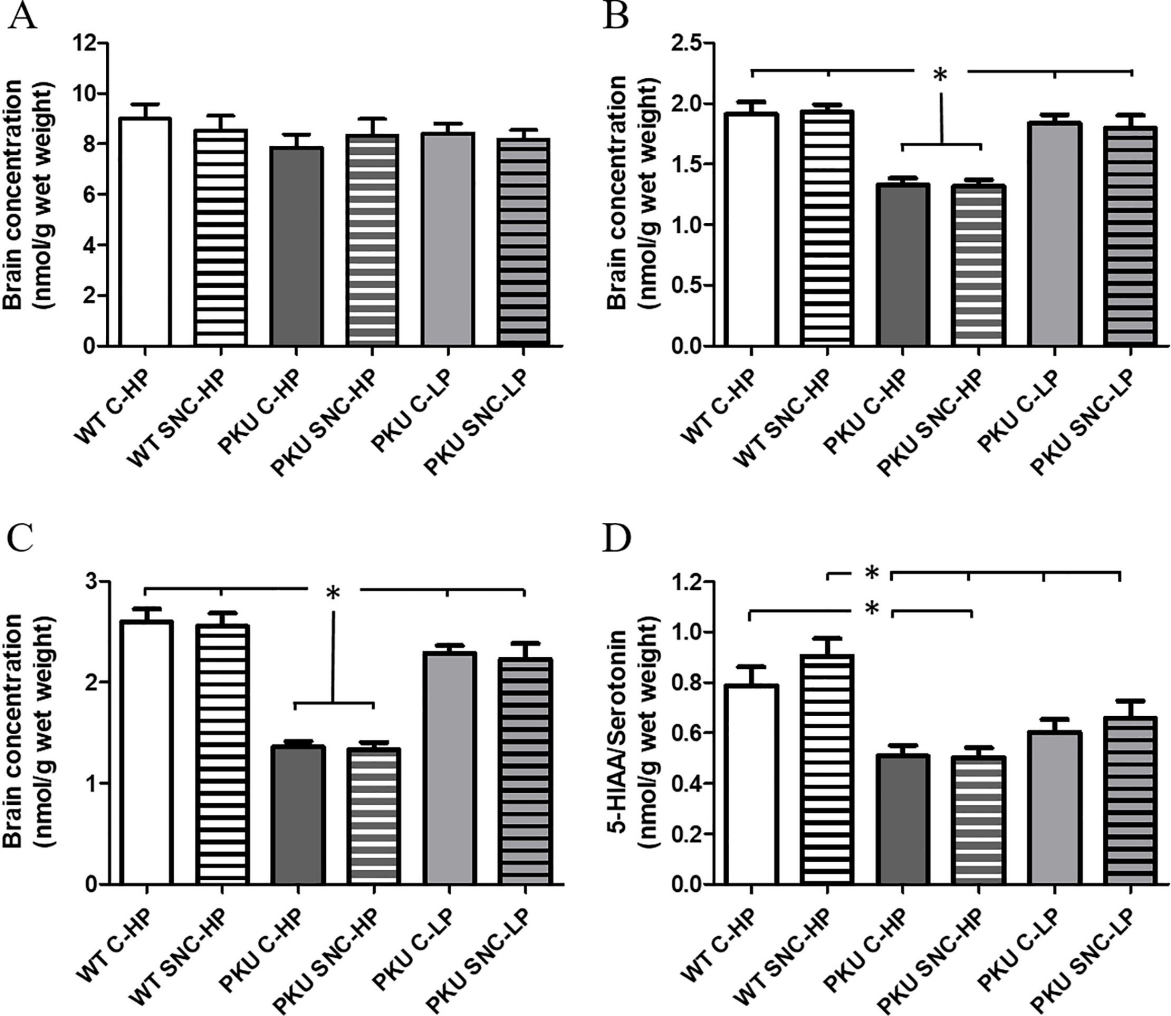
Neurotransmitter concentrations in brain. (A) Dopamine, (B) Norepinephrine, (C) Serotonin, (D) 5-HIAA/Serotonin; turnover of serotonin * p<0.05, 5-HIAA = 5-Hydroxyindoleacetic acid (metabolite of serotonin), n = 12, (mean ± SEM).

Next, we determined whether the behavioral differences between groups were associated with changes in neurotransmitter levels of the monoaminergic neurotransmitters dopamine, norepinephrine and serotonin in whole brain homogenates. No interaction effect was observed between sex and group (Dopamine: F(5,71) = 1.441, p = 0.223, Norepinephrine F(5,71) = 1.881, p = 0.111, Serotonin F(5,71) = 2.081, p = 0.080, or 5-HIAA/Serotonin F(5,71) = 1.042, p = 0.402). Therefore, Fig 8 depicts the data of both male and female mice.

As previously reported, PKU mice have lower levels of norepinephrine and serotonin, but not dopamine, in whole brain homogenates [17] (Fig 8: Dopamine F(5,72) = 0.526, p = 0.756, Norepinephrine F(5,71) = 14.737, p<0.001, Serotonin F(5,71) = 28.805, p<0.001, 5-HIAA/Serotonin F(5,71) = 8.009, p<0.001). In addition, serotonin turnover, measured as the ratio of NIAA/serotonin, was reduced in PKU mice compared to wild-type controls. Long lasting dietary treatment with a low Phe diet normalized these levels of norepinephrine and serotonin, but not serotonin turnover. However, despite having a positive effect on memory in the NOR, a long lasting diet with SNC did not affect the levels of any of the neurotransmitters measured.

For the turnover of serotonin (Fig 8D; 5-HIAA/serotonin), a significant difference was found between the WT C-HP and PKU C-HP and PKU SNC-HP (p = 0.013 and p = 0.011, respectively). WT on the supplemented diet had a higher turnover compared to all PKU groups (PKU C-HP p<0.001, PKU SNC-HP p<0.001, PKU C-LP p = 0.005, PKU SNC-LP p = 0.041). No significant differences were observed between the control and the corresponding supplemented groups for all depicted measurements.

## Discussion

In this study, a long-term dietary intervention with a low-Phe diet, a specific combination of nutrients designed to improve brain function or both concepts together was investigated in male and female BTBR PKU and WT mice. We replicated abnormalities in growth, motor behavior and learning and memory in PKU BTBR mice compared to WT BTBR controls, along with reduced levels of monoaminergic neurotransmitters in whole brain homogenate [12,17]. We found that both a long-lasting low Phe diet, the diet enriched with SNC as well as the combined diet was able to ameliorate some, but not all of these above mentioned PKU-induced abnormalities.

Specifically, a long-term low Phe diet, starting at the time of weaning, was able to almost completely normalize the brain levels of norepinephrine and serotonin, albeit without improving the serotonin turnover. At the same time, the low Phe diet improved growth and normalized performance on the balance beam, with the latter effect being more pronounced in male than in female mice. This is in line with the human situation where it is well established that early intervention in PKU patients can prevent the irreversible cognitive disabilities found in untreated PKU patients [18] and modify the outcome in those who are diagnosed at a later age. In our current study, we introduced the SNC supplementation and low-Phe conditions at PND 31. At this time point, the maturation of the brain and the characteristic behavior in mice is thought to represent the early adolescent stage in humans [19] and, in BTBR PKU mice, this time point is after the onset of the behavioral and neurochemical deficits [20]. The introduction of our nutritional intervention would, therefore, surpass the early prevention window of PKU treatment to prevent cognitive disabilities, which is inherent to the PKU mouse model where early intervention would be complex, as the pups continue to drink with their dam until weaning and any nutritional intervention to the pups would be highly stressful. Nevertheless, the PKU mice on a control low Phe diet did master the NOR paradigm, suggesting, at least in PKU mice, that the low Phe conditions introduced later in life can be beneficial for object recognition memory. These findings reinforce the notion that a life-long diet of protein restriction and amino acid substitutes is beneficial to PKU patients [18]. Indeed, even in late-diagnosed PKU patients, a Phe-restricted diet can still have positive effects [21].

The other dietary intervention that was tested in this study is a specific combination of nutrients (referred to as SNC) designed to improve brain function. These specific nutrients are precursors and cofactors for the synthesis of phospholipids through the Kennedy pathway [7]. Through increased phospholipid synthesis, we hypothesized that the SNC nutrients might improve neurotransmitter release, synaptic functioning, white matter integrity, and oxidative stress in the PKU brain. Most evidence supporting these hypotheses comes from work in animal models of different conditions [7]. In the C57Bl6 PKU mouse model we have previously shown that SNC normalizes PSD-95 immunostaining intensity in subregions of the hippocampus [6], suggesting an effect on synaptic functioning. Improved synaptic functioning in the hippocampus could lead to improvements in learning and memory. Memory performance was tested in the SOR and NOR in this study [15,22]. The BTBR mouse, especially at an older age, have difficulties mastering the task irrespective of having PKU. The dietary impact on SOR performance is therefore inconclusive. However, we found that BTBR PKU mice, despite being subjected to high Phe conditions, could master the NOR task on all three time points when supplemented with SNC, while those that did not receive SNC could not. However, additional brain regions such the perirhinal cortex, and other brain regions involved in visual, olfactory, and somatosensory perception are important in object recognition, and the consolidation, acquisition and retrieval of the memory necessary for mastering the NOR paradigm [15,23]. As such, the current dataset does not allow us to make any mechanistic links between synaptic functioning and behavioral performance. Besides synaptic functioning also a decrease in neuroinflammation by SNC could play a role [24], as enhanced neuroinflammation is often found in metabolic diseases including PKU [25,26]. In contrast to an improvement on memory performance with SNC, this supplementation did not consistently influence the motor performance in PKU mice. In the SOR and open field, one could argue that the window between PKU and WT mice was too small to be able to observe significant differences induced by any intervention.

Previously it was shown that acetylcholine release and signaling are positively affected by SNC supplementation [27,28]. Here we tested whether long-term SNC supplementation affects the whole brain levels of monoaminergic neurotransmitters that are lower in the PKU brain, most likely due to reduced availability of precursors because of competition of large neutral amino acids at the blood brain barrier [17]. However, while the low Phe diet normalized the levels of norepinephrine and serotonin in whole brain homogenate, SNC had no effect on these parameters.

This study was the first to investigate the long-term effect on behavior of different dietary interventions in the BTBR PKU mouse model. Therefore, we were unprepared for the loss of animals during the experiment, and notwithstanding that the deaths did not occur in specific groups, they led to unbalanced groups and smaller numbers of animals to be tested. As such, this study shows that future studies should be aware of this early drop-out of genetic models on a BTBR background. In this study, we performed multiple rounds of behavioral testing in the same animal which allows us to examine changes with aging. In the open field, we found a reduction in locomotor activity and an increase in time spent in the corners even in the WT mice, in addition to a difference between males and females. This reduced locomotor activity with aging is in line with other mouse studies that show this, although we cannot exclude the effect of habituation to the open field [29,30]. In the NOR and the balance beam, performance was stable over time with aging, which demonstrates the feasibility of retesting on the performance in these tasks. In contrast, in the SOR, the difference between WT and PKU mice observed at 3 months was not replicated at 6 and 9 months. Although it is possible that multiple rounds of behavioral testing could have influenced this outcome, impairments of spatial memory are reported in normal aging mice and BTBR mice [31–36]. However from these experiments it is not possible to conclude whether the lack of effect on the SOR at 6 and 9 months is due to aging or due to re-testing. Taken together, we have shown that it is possible to perform a long-term dietary intervention in the PKU mouse model, with reliable test-retest ability on different behavioral tasks, including NOR, open field, and balance beam.

## Conclusion

This study is the first to demonstrate that both a long-term low Phe diet, a diet enriched with SNC as well as both diets combined is able to ameliorate some, but not all of the PKU-induced abnormalities. Both diets seem to improve some, but not other domains that are impaired in the BTBR PKU mouse model. Specifically, this study demonstrates that a long-term intervention study in BTBR PKU mice improves novel object recognition, while a long-term intervention with a low Phe diet nearly normalizes serotonin and norepinephrine levels. Future research should be aimed at developing an optimal nutritional intervention to target brain function in PKU patients.

## Supporting information

S1 Table Overview of the mice that reached the humane endpoint or died before the end of the study. (DOCX)

## References

[1] Blau N, van Spronsen FJ, Levy HL. Phenylketonuria. *Lancet* 2010; 376: 1417–27. 10.1016/S0140-6736(10)60961-0 20971365

[2] Jahja R, van Spronsen FJ, de Sonneville LMJ, van der Meere JJ, Bosch AM, Hollak CEM et al Social-cognitive functioning and social skills in patients with early treated phenylketonuria: a PKU-COBESO study. *J Inherit Metab Dis* 2016; 39: 355–362. 10.1007/s10545-016-9918-0 26914933 PMC4851698

[3] Moyle JJ, Fox AM, Arthur M, Bynevelt M, Burnett JR. Meta-analysis of neuropsychological symptoms of adolescents and adults with PKU. *Neuropsychol Rev* 2007; 17: 91–101. 10.1007/s11065-007-9021-2 17410469

[4] Enns GM, Koch R, Brumm V, Blakely E, Suter R, Jurecki E. Suboptimal outcomes in patients with PKU treated early with diet alone: revisiting the evidence. *Mol Genet Metab*; 101: 99–109. 10.1016/j.ymgme.2010.05.017 20678948

[5] Palermo L, Geberhiwot T, MacDonald A, Limback E, Hall SK, Romani C. Cognitive outcomes in early-treated adults with phenylketonuria (PKU): A comprehensive picture across domains. *Neuropsychology* 2017; 31: 255–267. 10.1037/neu0000337 28080075 PMC5328133

[6] Bruinenberg VM, van Vliet D, Attali A, de Wilde MC, Kuhn M, van Spronsen FJ et al A Specific Nutrient Combination Attenuates the Reduced Expression of PSD-95 in the Proximal Dendrites of Hippocampal Cell Body Layers in a Mouse Model of Phenylketonuria. *Nutrients* 2016; 8 10.3390/nu8040185 27102170 PMC4848654

[7] van Wijk N, Broersen LM, de Wilde MC, Hageman RJJ, Groenendijk M, Sijben JWC et al Targeting synaptic dysfunction in Alzheimer’s disease by administering a specific nutrient combination. *J Alzheimers Dis* 2014; 38: 459–79. 10.3233/JAD-130998 23985420

[8] de Wilde MC, Penke B, van der Beek EM, Kuipers AA, Kamphuis PJ, Broersen LM. Neuroprotective effects of a specific multi-nutrient intervention against Aβ42-induced toxicity in rats. *J Alzheimers Dis*. 2011; 27:327–39. 10.3233/JAD-2011-110635 21811020

[9] Thau-Zuchman O, Gomes RN, Dyall SC, Davies M, Priestley JV, Groenendijk M, et al Brain Phospholipid Precursors Administered Post-Injury Reduce Tissue Damage and Improve Neurological Outcome in Experimental Traumatic Brain Injury. *J Neurotrauma*. 2019; 36:25–42. 10.1089/neu.2017.5579 29768974 PMC6306688

[10] Cansev M, van Wijk N, Turkyilmaz M, Orhan F, Sijben JW, Broersen LM. Specific multi-nutrient enriched diet enhances hippocampal cholinergic transmission in aged rats. *Neurobiol Aging*. 2015; 36:344–51. 10.1016/j.neurobiolaging.2014.07.021 25146455

[11] Wurtman RJ, Cansev M, Sakamoto T, Ulus IH. Use of phosphatide precursors to promote synaptogenesis. *Annu Rev Nutr* 2009; 29: 59–87. 10.1146/annurev-nutr-080508-141059 19400698

[12] Bruinenberg VM, van der Goot E, van Vliet D, de Groot MJ, Mazzola PN, Heiner-Fokkema MR et al The Behavioral Consequence of Phenylketonuria in Mice Depends on the Genetic Background. *Front Behav Neurosci* 2016; 10: 233 10.3389/fnbeh.2016.00233 28066199 PMC5167755

[13] Pascucci T, Giacovazzo G, Andolina D, Accoto A, Fiori E, Ventura R et al Behavioral and neurochemical characterization of new mouse model of hyperphenylalaninemia. *PLoS One* 2013; 8: e84697 10.1371/journal.pone.0084697 24376837 PMC3869930

[14] Hovens IB, Schoemaker RG, van der Zee EA, Absalom AR, Heineman E, van Leeuwen BL. Postoperative cognitive dysfunction: Involvement of neuroinflammation and neuronal functioning. *Brain Behav Immun* 2014; 38: 202–10. 10.1016/j.bbi.2014.02.002 24517920

[15] Antunes M, Biala G. The novel object recognition memory: neurobiology, test procedure, and its modifications. *Cogn Process* 2012; 13: 93–110. 10.1007/s10339-011-0430-z 22160349 PMC3332351

[16] van Vliet D, Bruinenberg VM, Mazzola PN, van Faassen MHJR, de Blaauw P, Kema IP et al Large Neutral Amino Acid Supplementation Exerts Its Effect through Three Synergistic Mechanisms: Proof of Principle in Phenylketonuria Mice. *PLoS One* 2015; 10: e0143833 10.1371/journal.pone.0143833 26624009 PMC4666635

[17] van Vliet D, Bruinenberg VM, Mazzola PN, van Faassen MH, de Blaauw P, Pascucci T et al Therapeutic brain modulation with targeted large neutral amino acid supplements in the Pah-enu2 phenylketonuria mouse model. *Am J Clin Nutr* 2016; 104: 1292–1300. 10.3945/ajcn.116.135996 27655443

[18] van Spronsen FJ, van Wegberg AM, Ahring K, Bélanger-Quintana A, Blau N, Bosch AM et al Key European guidelines for the diagnosis and management of patients with phenylketonuria. *Lancet Diabetes Endocrinol* 2017 10.1016/S2213-8587(16)30320-5 28082082

[19] Semple BD, Blomgren K, Gimlin K, Ferriero DM, Noble-Haeusslein LJ. Brain development in rodents and humans: Identifying benchmarks of maturation and vulnerability to injury across species. *Prog Neurobiol* 2013; 106–107: 1–16. 10.1016/j.pneurobio.2013.04.001 23583307 PMC3737272

[20] Fiori E, Oddi D, Ventura R, Colamartino M, Valzania A, D’Amato FR, Bruinenberg V, van der Zee E, Puglisi-Allegra S PT. Early-onset behavioral and neurochemical deficits in the genetic mouse model of phenylketonuria. *PLoS One* 2017; 12 10.1371/journal.pone.0183430 28850618 PMC5574541

[21] Koch R, Moseley K, Ning J, Romstad A, Guldberg P, Guttler F. Long-term beneficial effects of the phenylalanine-restricted diet in late-diagnosed individuals with phenylketonuria. *Mol Genet Metab* 1999; 67: 148–55. 10.1006/mgme.1999.2863 10356314

[22] Reger ML, Hovda DA, Giza CC. Ontogeny of Rat Recognition Memory measured by the novel object recognition task. *Dev Psychobiol* 2009; 51: 672–678. 10.1002/dev.20402 PMC2956740 19739136

[23] Oliveira AMM, Hawk JD, Abel T, Havekes R. Post-training reversible inactivation of the hippocampus enhances novel object recognition memory. *Learn Mem* 2010; 17: 155–160. 10.1101/lm.1625310 20189960 PMC2832924

[24] Wiesmann M, Zinnhardt B, Reinhardt D, Eligehausen S, Wachsmuth L, Hermann S, et al A specific dietary intervention to restore brain structure and function after ischemic stroke. *Theranostics*. 2017;7:493–512. 10.7150/thno.17559 28255345 PMC5327363

[25] van Dijk G, van Heijningen S, Reijne AC, Nyakas C, van der Zee EA, Eisel UL. Integrative neurobiology of metabolic diseases, neuroinflammation, and neurodegeneration. *Front Neurosci*. 2015; 9:173 10.3389/fnins.2015.00173 eCollection 2015. 26041981 PMC4434977

[26] van der Goot E, Bruinenberg VM, Hormann FM, Eisel ULM, van Spronsen FJ, Van der Zee EA. Hippocampal microglia modifications in C57Bl/6 Pah^enu2^ and BTBR Pah^enu2^ phenylketonuria (PKU) mice depend on the genetic background, irrespective of disturbed sleep patterns. *Neurobiol Learn Mem*. 2018 pii: S1074-7427(18)30113-8. 10.1016/j.nlm.2018.05.002 [Epub ahead of print] 29772389

[27] Cansev M, van Wijk N, Turkyilmaz M, Orhan F, Sijben JWC, Broersen LM. A specific multi-nutrient enriched diet enhances hippocampal cholinergic transmission in aged rats. *Neurobiol Aging* 2015; 36: 344–51. 10.1016/j.neurobiolaging.2014.07.021 25146455

[28] Savelkoul PJM, Janickova H, Kuipers AAM, Hageman RJJ, Kamphuis PJ, Dolezal V et al A specific multi-nutrient formulation enhances M1 muscarinic acetylcholine receptor responses in vitro. *J Neurochem* 2012; 120: 631–40. 10.1111/j.1471-4159.2011.07616.x 22146060

[29] Shoji H, Takao K, Hattori S, Miyakawa T. Age-related changes in behavior in C57BL/6J mice from young adulthood to middle age. *Mol Brain* 2016; 9: 11 10.1186/s13041-016-0191-9 26822304 PMC4730600

[30] Brouwer-Brolsma EM, Schuurman T, de Groot LCPMG, Feskens EJM, Lute C, Naninck EFG et al No role for vitamin D or a moderate fat diet in aging induced cognitive decline and emotional reactivity in C57BL/6 mice. *Behav Brain Res* 2014; 267: 133–143. 10.1016/j.bbr.2014.03.038 24680988

[31] Weber M, Wu T, Hanson JE, Alam NM, Solanoy H, Ngu H et al Cognitive Deficits, Changes in Synaptic Function, and Brain Pathology in a Mouse Model of Normal Aging. *eNeuro* 2015; 2 10.1523/ENEURO.0047-15.2015 26473169 PMC4606159

[32] Saroja SR, Kim E-J, Shanmugasundaram B, Höger H, Lubec G. Hippocampal monoamine receptor complex levels linked to spatial memory decline in the aging C57BL/6J. *Behav Brain Res* 2014; 264: 1–8. 10.1016/j.bbr.2014.01.042 24508236

[33] MacPherson P, McGaffigan R, Wahlsten D, Nguyen P V. Impaired fear memory, altered object memory and modified hippocampal synaptic plasticity in split-brain mice. *Brain Res* 2008; 1210: 179–88. 10.1016/j.brainres.2008.03.008 18417102

[34] Stapley NW, Guariglia SR, Chadman KK. Cued and contextual fear conditioning in BTBR mice is improved with training or atomoxetine. *Neurosci Lett* 2013; 549: 120–4. 10.1016/j.neulet.2013.06.032 23827222

[35] Mulder CK, Reckman GA, Gerkema MP, Van der Zee EA. Time-place learning over a lifetime: absence of memory loss in trained old mice. Learn Mem 2015; 22: 278–88. 10.1101/lm.037440.114 25903452 PMC4408771

[36] Van der Zee EA. Synapses, spines and kinases in mammalian learning and memory, and the impact of aging. Neurosci Biobehav Rev 2015; 50: 77–85. 10.1016/j.neubiorev.2014.06.012 24998408

