# Supplementary material for: Long-term dietary intervention with low Phe and/or a specific nutrient combination improve certain aspects of brain functioning in phenylketonuria (PKU)
Source: PLoS One. 2019 Mar 15;14(3):e0213391. doi: 10.1371/journal.pone.0213391 (PMC6420157; doi:10.1371/journal.pone.0213391)
Supplement: S1 Table — (DOCX) [file pone.0213391.s001.docx]

Supplementary Table 1

Overview of the mice that reached the humane endpoint or died before the end of the study.

| Group | Gender | Genotype | Age (mo) | Health issue and consequence |
| --- | --- | --- | --- | --- |
|  | | | | |
| C-LP | Female | PKU | 3.7 | cervical prolapse; euthanized |
| C-LP | Female | PKU | 6.9 | blood in mouth; found dead |
|  |  |  |  |  |
| C-HP | Female | PKU | 3.1 | cervical prolapse; euthanized |
| C-HP | Male | WT | 4.7 | enlarged heart; euthanized |
| C-HP | Female | PKU | 4.8 | unusual large stomach (filled); found dead |
|  | | | | |
| F-LP | Male | PKU | 5.6 | unclear; found dead |
|  | | | | |
| F-HP | Female | PKU | 1.5 | developed elephant teeth; euthanized |
| F-HP | Female | PKU | 2.2 | cervical prolapse/teeth problems; euthanized |
| F-HP | Female | PKU | 2.4 | abdomal bleeding; found dead |
| F-HP | Female | PKU | 4.6 | cervical prolapse; euthanized |
| F-HP | Female | PKU | 5.4 | blockage in the eusophagus; euthanized |
| F-HP | Male | PKU | 8.1 | kidney failure; found dead |
| F-HP | Male | WT | 8.1 | small piece of intestine was enlarged; found dead |
| F-HP | Male | WT | 9.0 | kidney failure; found dead |
| F-HP | Male | WT | 9.3 | kidney failure; found dead |
